# Epidemic Spread of Lyme Borreliosis, Northeastern United States

**DOI:** 10.3201/eid1204.051016

**Published:** 2006-04

**Authors:** Klára Hanincová, Klaus Kurtenbach, Maria Diuk-Wasser, Brandon Brei, Durland Fish

**Affiliations:** * Yale University, New Haven, Connecticut, USA

**Keywords:** zoonoses, Lyme borreliosis, spread, ticks, host specialization, research

## Abstract

We examined the degree of host specialization of different strains of *Borrelia burgdorferi*, the tickborne pathogen that causes Lyme borreliosis in the northeastern United States. We first assessed the genetic population structures of *B. burgdorferi* in ticks obtained from different mammalian host species and in questing ticks sampled in a woodland ecosystem in Connecticut. By comparing the patterns found in our study with data from another cross-sectional study, we demonstrate that *B. burgdorferi* is a generalist microparasite and conclude that efficient cross-species transmission of *B. burgdorferi* is a key feature that has allowed the rapid spread of Lyme borreliosis across the northeastern United States.

## Abstract 2

Host specialization is a key issue in infectious disease research because patterns of cross-species transmission affect parasite dispersal.

The evolution of specialization remains a major problem in ecology and evolutionary biology; why some species are generalists and others are specialists is not resolved (1,2). Like all organisms, parasites have evolved to different levels of ecologic specialization (3–5). The level of host specialization of parasites is a key issue in infectious disease research because patterns of cross-species transmission affect parasite dispersal and can facilitate epidemics. West Nile virus is a recent example illustrating that the utilization of many highly mobile host species can enable a pathogen to disperse across an entire continent within a few years (6). Multihost parasites are usually considered to be generalists; however, this is not universally true, and several examples exist in which generalist parasites are structured into subpopulations that are host specialized (7). Theory predicts that natural selection favors host specialization if hosts are abundant and predictable, whereas generalist strategies evolve if hosts are erratic (8).

*Borrelia burgdorferi*, the spirochetal agent of Lyme borreliosis (LB) in the United States, is a tickborne zoonotic pathogen that infects an expansive range of vertebrate species, involving diverse mammalian and avian hosts (9–14). For this reason, it has been suggested *B. burgdorferi* is likely less specialized than the other genospecies that cause LB in Eurasia (15–18). Several loci of *B. burgdorferi* are polymorphic (19), and balancing selection seems to maintain the bacterium's diversity (20). Given the pronounced strain structure of this bacterial species, natural selection possibly has driven *B. burgdorferi* towards host specialization, and different spirochete strains exploit different sets of vertebrate hosts (4,13).

The issue of vertebrate host specialization of *B. burgdorferi* is of substantial public health importance. Since the reemergence of LB 3 decades ago, the disease has been spreading across the entire northeastern United States and beyond (21,22). A condition necessary for this dispersal has been the geographic expansion of its principal and generalist tick vector, *Ixodes scapularis*. This expansion is believed to be driven by large-scale reforestation and an explosive growth of deer populations (21). Deer, however, do not contribute directly to the dispersal of *B. burgdorferi* (23). Only hosts that can infect ticks affect spirochete migration. If *B. burgdorferi* were host specialized, the strains of this microparasite would migrate differentially, resulting in geographic structuring of this pathogen. Unrestricted cross-species transmission, in contrast, would generate a spatially uniform population structure of *B. burgdorferi* and substantially facilitate its dispersal. Information on the level of host specialization of this multihost pathogen is required to understand the patterns and mechanisms of the current spread of LB.

We examined the level of host specialization of *B. burgdorferi* in the northeastern United States by using a comparative approach. We first assessed the genetic population structures of *B. burgdorferi* in ticks obtained from different mammalian host species and in questing ticks sampled in a woodland ecosystem at Lake Gaillard, Branford, Connecticut. By comparing the patterns in our study with data from another cross-sectional study carried out in a similar ecosystem in Millbrook, New York (13), we aimed to capture patterns of cross-species transmission and to identify the niche breadth of the various genotypes of *B. burgdorferi*.

## Materials and Methods

### Mammal Sampling

The fieldwork was carried out at Lake Gaillard (41°34´N, 72°77´W), Connecticut, as described previously (24). Mammals were captured alive at 2-week intervals from early June until late August in 2002 and until mid-September in 2003. All trapping and handling procedures were approved by the Yale University Institutional Animal Care and Utilization Committee (Study Protocol 07596). Small mammals were trapped for 23 days/nights (432 trap nights) by using Sherman (Tallahassee, FL, USA) traps. In addition, Pitfall traps were set up for 14 days/nights (98 trap nights) in 2003. Medium-sized mammals were captured for 27 days/nights (820 trap nights) and 25 days/nights (724 trap nights) by using Tomahawk (Tomahawk, WI, USA) trap numbers 205 and 207, respectively. All captured mammals were housed over pans of water for 72 hours to recover engorged ticks. Ticks were allowed to molt to the next developmental stage, determined to species, and stored in 70% ethanol. Mammals were marked, sexed, and measured. Before handling, mammals were anesthetized with ketamine hydrochloride or a combination of ketamine hydrochloride and xylazine. After captivity, mammals were released at their original location.

### Host-seeking Ticks

Questing *I. scapularis* nymphs were collected over the same period and in the same area where the mammals were captured by dragging the vegetation with 1-m^2^ drag cloths. Collected ticks were preserved in 70% ethanol.

### DNA Extraction and PCR

DNA was extracted from ticks according to a DNeasy Tissue Kit protocol (Qiagen, Valencia, CA, USA) as described previously (25). Ticks were screened for *B. burgdorferi* DNA by real-time Taqman polymerase chain reaction (PCR) targeting the 16S rDNA of *B. burgdorferi* (24). Positive samples were then subjected to nested PCR amplifying a fragment of the *rrs* (16S)–*rrl* (23S) intergenic spacer of *B. burgdorferi* and sequenced (19).

### Data Analysis

Infectivity of hosts to ticks was determined by identifying *B. burgdorferi* in molted nymphs derived from mammals. Since transovarial transmission of *B. burgdorferi* to larval *I. scapularis* has not been demonstrated, infections found in molted nymphs were assumed to be acquired from a host through feeding. A mammal, therefore, was considered infectious to ticks if >1 nymphs that had fed, as larva, on that mammal tested positive.

To evaluate the exposure of animals to infected nymphs for each host species, the attachment rate of nymphs per animal per day (*RD_S_*) was computed for each capture time point as *RD_S_* = *A*/*F*; *A* is the mean number of feeding ticks per host and *F* is the average feeding time of *I. scapularis* nymphs, which was conservatively assumed to be 5 days (26). The minimum attachment rate of nymphs per animal per season (*RS_S_*) was computed as *RS_S_* = Σ (*RD_S_* × *C*); *C* is the number of days between capture points. The number of nymphs infected with a genotype encountered by a host per season (*RSI_S_*) was calculated as *RSI_S_* = *IP*/*N* × *RS_S_*; *IP* is the infection prevalence of a genotype in field-collected questing nymphs, and *N* is the number of nymphs tested. Since no data for May were obtained empirically, we extrapolated the data on nymphal infestation obtained at the end of peak activity (i.e., end of June) and applied it to May. This provided a conservative estimate of the total number of infected nymphs a host encountered over the nymphal activity season.

### Statistical Analysis

Differences in mean numbers of ticks per host were examined by using the nonparametric Kruskal-Wallis test. Logistic regression was used to estimate the infection prevalence in ticks or hosts and to compare them among host species. Presence of *B. burgdorferi* infection in a tick or host was the response variable in the model, and a dummy variable for host species was used as the predictor. The advantage of using logistic regression models for proportional data are that different coding systems can be applied to compare infection prevalence among various groupings of host species (e.g., mice vs. other hosts). Additionally, logistic models can control for the fact that several ticks were collected from the same mammal and were not independent samples. In this analysis, a cross-sectional procedure (Stata xtlogit) was applied to control for the correlation among ticks collected from the same mammal (27). To test for a sample size effect on the number of genotypes found in a host species, a Spearman rank correlation was performed between the number of genotypes and the number of mammals sampled for each host species. The differences in genotype frequency distributions were estimated through exact nonparametric inference by the Fisher-Freeman-Halton test (Monte Carlo testing). Pearson's χ^2^ test was used to compare the proportions of ticks infected with different genotypes within and among host species. Data were analyzed with Stata, version 8, (Stata Corporation, College Station, TX, USA) and StataXact, version 6, (Cytel, San Diego, CA, USA).

## Results

### Mammal Trapping

Sampling over 2 years yielded 403 captures that included 222 individual mammals, representing 9 mammalian species of 6 families (*Muridae*, *Soricidae*, *Sciuridae*, *Mustelidae*, *Procyonidae*, and *Didelphiidae*) belonging to 4 orders (Rodentia, Insectivora, Carnivora, and Marsupialia). Six species (white-footed mouse, pine vole, eastern chipmunk, gray squirrel, Virginia opossum, and raccoon) accounted for 98% of all mammals caught (Table 1).

**Table 1:** Captured mammals and their infestation with *Ixodes scapularis* *

Host species	No. hosts	No. captures	Larvae†		Nymphs‡	
			N	Mean (SE)	N	Mean (SE)
White-footed mouse (*Peromyscus leucopus*)	132	283	2,548	9.0 (0.7)	414	1.5 (0.2)
Pine vole (*Microtus pinetorum*)	23	23	127	5.5 (2.3)	17	0.7 (0.2)
Eastern chipmunk (*Tamias striatus*)	3	8	106	13.3 (7.3)	145	18.1 (13.0)
Gray squirrel (*Sciurus carolinensis*)	14	22	117	5.3 (1.3)	321	14.6 (4.8)
Raccoon (*Procyon lotor*)	39	49	3,630	77.0 (14.5)	394	8.0 (1.1)
Virginia opossum (*Didelphis virginiana*)	7	14	1,083	77.4 (25.2)	82	5.9 (3.3)
Common shrew (*Sorex cinereus*)	1	1	NA		NA	
Short-tailed shrew (*Blarina brevicauda*)	2	2	NA		NA	
Stripped skunk (*Mephitis mephitis*)	1	1	NA		NA	

*SE, standard error; NA, not analyzed. †Kruskal-Wallis test, χ^2^ = 33.61, degree of freedom (df) = 5; p<0.001. ‡Kruskal-Wallis test, χ^2^ = 57.76, df = 5; p<0.001.

### Tick Infestation

Altogether, 9,032 immature ticks were collected from 399 captured hosts. The most abundant tick species, *I. scapularis*, represented 99% (7,611 larvae and 1,373 nymphs) of all ticks examined. The additional 3 species, *I. texanus*, *Dermacentor variabilis*, and *Amblyomma maculatum*, comprised the remaining 1% and were omitted from further analysis. The mean numbers of *I. scapularis* ticks per host varied significantly among mammalian species for both larvae and nymphs (Table 1).

### *B. burgdorferi* Prevalence in Host-derived Ticks

Of the nymphs sampled from 62 mammals as engorged larvae, 1,117 specimens were screened for presence of *B. burgdorferi*. The number of tested nymphs per host varied from 1 to 51, depending mainly on the number of engorged larvae recovered. *B. burgdorferi*–positive ticks were obtained from all 6 mammalian species.

Infection prevalence of *B. burgdorferi* in animals varied significantly among host species (logistic regression, χ^2^ =14.15, p<0.01) (Table 2). Each of the 3 tested chipmunks produced >1 infected nymphs and, therefore, this species was excluded from the logistic regression model, since the presence of a zero category (noninfectious chipmunks) produced infinite odds ratios (OR), which precluded the estimation of the model. No significant differences were found between voles, squirrels, raccoons, and opossums. Hence, these species were pooled and compared with mice. The proportion of infectious mice was significantly higher than that of the pooled group of other host species (logistic regression, OR 13.42, 95% confidence interval (CI) 1.63–110.41, p<0.001).

**Table 2:** Proportions of infectious hosts*

Host species	No. infectious/tested hosts (%)	No. infectious hosts†								
		GT1	GT2	GT3	GT4	GT5	GT6	GT7	GT8	MI
White-footed mouse	14/15 (93.3)	3	8	10	2	6	1	1	3	11
Pine vole	9/17 (52.9)	4	4	1	1	1		1		2
Eastern chipmunk	3/3 (100)		1			1			2	1
Gray squirrel	4/10 (40.0)				3				3	1
Raccoon	3/10 (30)	1	2			1		1		1
Virginia opossum	4/6 (66.7)	1		3	1	1				2
Total	37/62 (59.8)	9	15	14	7	10	1	3	8	18

*GT, genotype; MI, mixed infections. †In some cases, the sum of genotype infections was greater than the number of infected ticks because of mixed infections.

Infection prevalences of *B. burgdorferi* in host-derived ticks also varied significantly among host species (logistic regression, χ^2^ = 42.38, p<0.001) (Table 3). A considerably higher infection prevalence in ticks was observed for mice than for voles (logistic regression, OR 16.37, 95% CI 4.73–56.69, p<0.001). On the other hand, no significant differences in tick infection prevalence were found among raccoons, opossums, squirrels, and chipmunks. Therefore, data for these host species were pooled into 1 group. Infection prevalence in ticks from mice was significantly higher than in ticks from the pooled group (logistic regression, OR 47.89, 95% CI 14.97–153.23, p<0.001), as was infection prevalence in ticks from voles compared to the pooled group (logistic regression, OR 2.92, 95% CI 1.16–7.34, p<0.001).

**Table 3:** *Borrelia burgdorferi* infections and frequency distributions of genotypes in ticks*

Host species	No. of infected/tested ticks (%)	No. of ticks infected with different genotypes (%)†								
		GT1	GT2	GT3	GT4	GT5	GT6	GT7	GT8	ND
White-footed mouse	90/100 (90.0)	11 (11.0)	31 (31.0)	26 (26.0)	4 (4.0)	10 (10.0)	2 (2.0)	1 (1.0)	4 (4.0)	11 (11.0)
Pine vole	29/113 (25.7)	13 (11.5)	7 (6.2)	1 (0.9)	2 (1.8)	1 (0.9)		1 (0.9)		5 (4.4)
Eastern chipmunk	17/73 (23.3)		1 (1.4)			4 (5.5)			12(16.4)	
Gray squirrel	19/60 (31.7)				16 (26.7)				7 (11.7)	
Raccoon	38/500 (7.6)	5 (1.0)	5 (1.0)			15 (3.0)		8 (1.6)		5 (1.0)
Virginia opossum	27/271 (10.0)	1 (0.4)		13 (4.8)	3 (1.1)	1 (0.4)				5 (1.8)
Subtotal	220/1,117 (19.7)	30 (2.7)	44 (3.9)	40 (3.6)	25 (2.2)	31 (2.8)	2 (0.2)	10 (0.9)	23 (2.1)	25 (2.2)
Field-collected nymphs	69/178 (38.8)	8 (4.5)	33 (18.5)	17 (9.6)	6 (3.4)	5 (2.8)	2 (1.1)	1 (0.6)	3 (1.7)	

*GT, genotype; ND, not determined. †In some cases, the sum of genotype infections was greater than the number of infected ticks because of mixed infections (data not shown).

### Population Structure of *B. burgdorferi* in Host-derived Ticks

A total of 205 *B. burgdorferi* infections in nymphs obtained from mammals as engorged larvae could be sequenced successfully. The IGS alleles were assigned to previously identified multilocus genotypes (19), designated here as genotypes 1 to 9. A total of 8 genotypes was shown (Tables 2,3, and 4). The white-footed mouse was the only host species that transmitted all 8 genotypes to ticks. None of the genotypes was transmitted by all host species. However, genotypes 1–5 and 7 were found in ticks collected from as many as 5 host species belonging to 3 different orders (Rodentia, Carnivora, Marsupialia). Only mice were found to be infectious for genotype 6. No significant relationship was found between the number of genotypes and the number of sampled individuals of a host species (Spearman rank correlation, *r_s_* = 0.5, p>0.05). The frequency distribution of transmitted genotypes differed significantly among host species (6 × 8 Fisher-Freeman-Halton test, Fisher statistic = 41.93; Monte Carlo p 0.05) (Table 2). The average number of genotypes per infectious host was 2.4 (standard error [SE] = 0.3) for mice, 1.7 (SE = 0.3) for opossums, 1.7 (SE = 0.7) for raccoons, 1.5 (SE = 0.5) for squirrels, 1.3 (SE = 0.2) for voles, and 1.3 (SE = 0.3) for chipmunks.

**Table 4:** Transmissibility of *Borrelia burgdorferi* genotypes from infectious hosts to ticks*

Host species	No. infected/ tested ticks (%)									
	GT1	GT2	GT3	GT4	GT5	GT6	GT7	GT8	Pearson χ^2^ (df)	p value
White-footed mouse	11/21 (52)	31/56 (55)	26/65 (40)	4/14 (29)	10/42 (24)	2/7 (3)	1/7 (14)	4/21 (19)	18.33 (7)	<0.01
Pine vole	13/25 (52)	7/52 (14)	NC	NC	1/7 (14)		1/7 (14)		14.56 (3)	<0.01
Eastern chipmunk		1/14 (7)			4/14 (29)			12/59 (20)	2.12 (2)	>0.05
Gray squirrel				16/26 (62)				7/32 (22)	9.43 (1)	<0.01
Raccoon	5/50 (10)	5/99 (5)			15/49 (31)		8 /51 (16)		19.36 (3)	<0.001
Virginia opossum	1/50 (2)		13/126 (10)	3/27 (11)	1/50 (2)				6.51 (3)	>0.05
Pearson χ^2^ (df)	45.17	46.94	22.97	15.20	15.16	NC	0.02	0.07		
p value	<0.001	<0.001	<0.001	0.001	<0.01	NC	>0.05	>0.05	6.51 (3)	>0.05

*GT, genotype; df, degrees of freedom;NC, not calculated.

The frequency distributions of genotypes in host-derived ticks are shown in Table 3. In most of the ticks obtained from mice, genotype 2 was identified, followed by genotype 3. Most of the ticks that had fed on voles were found to carry genotype 1. On the other hand, genotypes 8 and 4 were the most frequently detected variants in ticks obtained from chipmunks and squirrels, respectively. Genotype 5 was the most common genotype found in ticks derived from raccoons, and genotype 3 was the most frequent in ticks obtained from opossums.

Transmissibility of each genotype from infectious mammals to ticks can be regarded as a fitness index of strains infecting hosts. The values varied significantly within and among host species as shown in Table 4.

### *B. burgdorferi* in Field-collected Questing Nymphs and Exposure of Animals

A total of 178 field-collected questing nymphs were screened for *B. burgdorferi*. The overall infection prevalence was 39%. In this tick population, the same 8 genotypes as in nymphs derived from the animal pool were found. However, significant differences in the genotype frequency distribution between these 2 tick populations were observed (2 × 8 Fisher-Freeman-Halton, Fisher statistic = 19.93, Monte Carlo p<0.01) (Table 3). Questing nymphs were chosen to estimate the exposure of hosts to *B. burgdorferi* genotypes. The calculated values of exposure show that animals with higher nymphal burdens were frequently exposed to >1 infected nymph. This value was occasionally <1, reflecting the conservative estimation of exposure (Table 5).

**Table 5:** Exposure of animals to infected nymphs*

Host species	No. infected nymphs per host†							
	GT1	GT2	GT3	GT4	GT5	GT6	GT7	GT8
White-footed mouse	2.2	9.7	5.7	1.1	1.7	0.29	0.4	0.4
Pine vole	0.8	2.2	0.7	0.3	0.2	0.3	0	0.5
Eastern chipmunk	9.1	45.5	29.6	4.6	9.1	0	2.3	0
Gray squirrel	12.6	58.0	34.3	6.3	10.4	1.5	2.4	2.3
Raccoon	6.5	25.4	12.2	3.2	3.5	2.0	0.6	2.9
Virginia opossum	9.0	29.1	9.0	4.5	2.2	4.5	0	6.7

*GT, genotype. †For mice, raccoons and squirrels, calculated values are given as average numbers per season for years 2002 and 2003. For other species, average numbers are given per season for year where data were available.

### Comparative Analysis of *B. burgdorferi* Population Structures

Only 1 other study has analyzed the population structures of *B. burgdorferi* in different vertebrate host species in the northeastern United States (13). As in our study, transmissible infections in hosts were determined through host-derived ticks. In contrast, the population structures of *B. burgdorferi* were measured at the outer surface protein *C* (*ospC*) locus. However, because the *ospC* locus and the IGS used in our study are linked (19), the population structures found in both studies may be compared. Genotypes 1–8 were present in questing nymphs in each study, which indicates that the host populations were exposed to a similar spectrum of spirochete strains. Three rodent species, white-footed mice, chipmunks, and squirrels, were captured in both studies and used for comparison. The population structures of *B. burgdorferi* in each host species were different in the 2 data sets. However, analysis of the combined data set shows that, with the exception of genotypes 6 and 7 being missing in both squirrel populations, all 9 major genotypes that were prevalent in questing ticks were also found in the 3 rodent species (Table 6).

**Table 6:** *Borrelia burgdorferi* genotypes transmitted by hosts and detected in field-collected ticks in 2 ecosystems

Host species	Locality*	Genotype (GT) (major *ospC* group)†								
		GT1 (A)	GT2 (K)	GT3 (B)	GT4 (N)	GT5 (D)	GT6 (M)	GT7 (I)	GT8 (U)	GT9 (E)
White-footed mouse	Millbrook	+	+	+	+	+	+	+	–	+
	Lake Gaillard	+	+	+	+	+	+	+	+	–
	Combined	+	+	+	+	+	+	+	+	+
Eastern chipmunk	Millbrook	+	+	+	+	+	+	+	+	+
	Lake Gaillard	–	+	–	–	+	–	–	+	–
	Combined	+	+	+	+	+	+	+	+	+
Gray squirrel	Millbrook	+	+	+	–	+	–	–	–	+
	Lake Gaillard	–	–	–	+	–	–	–	+	–
	Combined	+	+	+	+	+	–	–	+	+
Field-collected nymphs	Millbrook	+	+	+	+	+	+	+	+	+
	Lake Gaillard	+	+	+	+	+	+	+	+	–

*Millbrook data based on (13); Lake Galliard data based on our observation. †+ indicates presence of genotype; – indicates absence of genotype.

## Discussion

We explored the question of whether, and to what level, *B. burgdorferi* is specialized to vertebrate host species. By analyzing 2 independent data sets obtained from cross-sectional field studies in the northeastern United States (New York and Connecticut), we show that most of the known genotypes of *B. burgdorferi* can, in principle, infect a range of different rodent hosts. Furthermore, our own data set indicates that several genotypes can infect as many as 5 host species. This suggests that cross-species transmission of *B. burgdorferi* among various mammalian species is common.

Several issues, however, need to be addressed before the level of host specificity of *B. burgdorferi* can be confidently compared with that of other microparasites. First, the level of host specificity is generally dependent on the spatial and temporal scale of observation (1,28). Our findings exemplify this notion, because the combined data sets of the 2 field surveys analyzed in this study show a pattern of more relaxed host specificity than each of the data sets would suggest on its own. Second, the "niche breadth" of a parasite is influenced by the phylogenetic relationships of its hosts (29). If, for example, a parasite infects a given number of hosts belonging to different orders or classes, one would consider such a parasite to be less specialized than a parasite that exploits the same number of closely related species.

The analysis of the combined data sets obtained by the 2 field surveys compared in this study shows that mice, chipmunks, and squirrels (order Rodentia) are susceptible to most of the *B. burgdorferi* genotypes described in the United States. Therefore, the niche breadth of *B. burgdorferi* genotypes is not congruent with host species. Furthermore, genotypes 1–5 and 7 can, at least transiently, infect many additional, phylogenetically distant host species, covering as many as 3 orders. This indicates that the niche breadth of most *B. burgdorferi* genotypes in the United States is even wider than the taxonomic unit of order.

The issue of host specificity in *B. burgdorferi*, however, is more complicated. Experimental work has shown that some isolates of *B. burgdorferi* do not disseminate, or slowly disseminate, in mice (30). Slowly disseminating strains are less efficiently transmitted to ticks by mice (31). For this reason, it has been suggested that such strains may occupy nonrodent or even nonmammalian niches in nature, such as avian hosts (31). On the other hand, certain strains of *B. burgdorferi* can infect both rodent and avian hosts (10), which demonstrates that some strains of *B. burgdorferi* are extreme generalists. In view of all ecologic and experimental information available to date, we conclude that host specificity of *B. burgdorferi* ranges from generalism to specialism, depending on genetic background.

Several possible explanations exist for the discordance between the data sets from New York and Connecticut. First, differences in the local ecologic conditions could shape the local population structures of *B. burgdorferi* in hosts (1). Furthermore, the 2 data sets could represent snapshots of population structures that are spatially and temporally variable due to stochastic effects or other forces (32). In other words, the spirochete populations could be dynamic. In fact, strong evidence exists for this scenario, since pronounced temporal shifts in genotype frequency distribution of *B. burgdorferi* within 2 years have been observed in questing adult ticks (33). Considering that adult *I. scapularis* ticks have a history of taking only 2 blood meals in 2 years (26), the scale of this temporal variation is remarkable.

One of the most fundamental parameters in infectious disease biology is the time scale of infectivity relative to host lifetime, which affects the epidemic/endemic behavior of all microparasites (34). *B. burgdorferi* infections in mice are believed to be lifelong (35). The universality of this paradigm, however, has recently been challenged by experimental studies in white-footed mice, which found that the infectivity of some strains to ticks declines within a few weeks (31,36). This feature is crucial in 2 ways. First, it indicates that fitness of *B. burgdorferi* is a quantitative trait. This is corroborated by our study that provides ample evidence for fitness variation within and across diverse host species. Second, the finding of declining infectivity shows that the transmission kinetics of some *B. burgdorferi* strains is dynamic. Therefore, both intrinsic transmission dynamics of *B. burgdorferi* strains in hosts (37) and population fluctuations of the hosts (38) may result in population fluctuations of *B. burgdorferi*. Time series analyses of spirochete populations are required to clarify the scale of the spatiotemporal dynamics of *B. burgdorferi* (32).

We are beginning to understand key molecular processes that enable cross-species transmission of *B. burgdorferi* (11,16). Individual strains of *B. burgdorferi* have been found to contain large arrays of prophage-encoded outer surface proteins that differentially bind complement control factors of a wide range of vertebrate species, preventing the bacteria from being killed by innate immunity (11). That the repertoire of these prophage genes determines the host range of LB spirochetes has been hypothesized (11,16). *B. burgdorferi*, thus, is 1 of the very few examples of zoonotic pathogens for which a molecular mechanism of host-switch has been proposed (39).

OspA serotypes 2–8, which comprise the Eurasian genospecies *B. afzelii* and *B. garinii*, occupy distinct host niches, such as rodent versus avian hosts (16). Here we demonstrate that *B. burgdorferi* (OspA serotype 1) in the northeastern United States is much less specialized than *B. afzelii* (serotype 2) and *B. garinii* (serotypes 3–8), because the niche breadth of most of its genotypes covers a much larger range of phylogenetically distant hosts than any of the other OspA serotypes. The generalist strategy of *B. burgdorferi* is consistent with its uniform population structure across much of the northeastern United States (33). We may speculate that the generalist strategy of *B. burgdorferi* echoes adaptation to impoverished ecologic conditions in the past because of large-scale habitat destruction in the northeastern United States in the course of the post-Columbian settlement and during the industrial revolution (8). We conclude that cross-species transmission of *B. burgdorferi* is a key property that has allowed LB to spread rapidly across the northeastern United States. Our study emphasizes that accurate information on the degree of cross-species transmission is necessary to understand and predict the spread of zoonotic pathogens.
